# Introducing the 45-year special collection: Driving innovation in primary care scholarship

**DOI:** 10.4102/safp.v68i2.6298

**Published:** 2026-03-05

**Authors:** Klaus B. von Pressentin, Ramprakash Kaswa, Simon M. Marcus, Mmaphefo M. Maluleka, Arun Nair, Mareike Rabe, Indiran Govender

**Affiliations:** 1 Division of Family Medicine, Department of Family, Community and Emergency Care, Faculty of Health Sciences, University of Cape Town, Cape Town, South Africa; 2 Department of Family Medicine & Rural Health, Faculty of Health Sciences, Walter Sisulu University, Mthatha, South Africa; 3 Department of Family Medicine, Faculty of Health Science, University of the Witwatersrand, Johannesburg, South Africa; 4 Department of Family Medicine and Primary Healthcare, Faculty of Medicine, Sefako Makgato Health Sciences University, Pretoria, South Africa; 5 Department of Family Medicine, University of the Free State, Kimberley, South Africa; 6 Vita Oncology, Cape Town, South Africa; 7 School of Rural Medicine, Charles Sturt University, New South Wales, Australia

Throughout its history, the *South African Family Practice* (SAFP) journal’s mission has been anchored in three interconnected pillars: advancing scientific knowledge, fostering academic growth and promoting social accountability. An editorial review of SAFP’s original research output (2020–2022) demonstrates the journal’s broad scope, covering clinical care, service delivery, health systems and educational research, with a significant proportion of student-driven studies, reflecting a maturing, context-aware research pipeline.^1^

This special collection in Issue 2, features a write-up of our ‘SAFP at 45’ panel discussion from the 27th National Family Practitioners Congress. The historical overview concluded at my appointment as editor-in-chief in March 2019, alongside Prof. Indiran Govender as senior assistant editor. With this editorial, we continue the story by reflecting on the journal’s current state and exploring future opportunities for SAFP in a rapidly evolving scientific publishing landscape.

Since transitioning to AOSIS as the publisher in 2020, we have adopted a fully online, ebook-compatible format and ensured that our journal policies align with international editorial standards, including those set by the Committee on Publication Ethics (COPE).^2,3^ We expanded our editorial team by appointing additional assistant editors, marking a shift from a solo-editor model to a dynamic, representative team focused on shared decision-making, inclusive leadership and building capacity for future succession. Assistant editors play an essential role in the peer-review process, but their primary focus is on shaping the journal’s Continuing Professional Development (CPD) content and promoting growth through collaboration with South African Academy of Family Physicians (SAAFP) interest groups. Their wide-ranging expertise – spanning oncology, emergency medicine and other clinical domains across both public and private sectors – enriches the journal’s breadth and depth.

The journal’s diverse editorial board (see https://safpj.co.za/index.php/safpj/pages/view/editorial-team) provides strategic guidance, drawing representation from the public and private sectors, all 10 academic departments and their family medicine registrars, as well as the Rural Doctors Association of South Africa (RuDASA). International experts in family medicine and primary care further enrich the board’s insights. Enhanced usage metrics, increased indexing visibility and closer monitoring of the review process have equipped the editorial team to manage both pace and quality more effectively. Regular collaboration with the publisher – including biweekly meetings with the submissions manager – ensures strong operational alignment. The publisher also delivers performance data and ongoing support, informing strategic discussions at the biannual editorial board meetings.

From the era of print advertising to today’s open-access, article processing charge (APC)-funded model, editorial independence remains SAFP’s cornerstone safeguard. The AOSIS publisher website highlights transparent policies regarding artificial intelligence (AI)-assisted technologies – emphasising disclosure and accountability – as part of its ongoing commitment to integrity.^4^ Governance practices such as COPE standards, authorship criteria, disclosure requirements, plagiarism checks and clear role separation are explicitly detailed on the journal’s website.

The open-access movement has further strengthened the journal’s dedication to equity by removing barriers for readers and expanding opportunities for authorship across diverse institutions and contexts. *South African Family Practice* supports authors and reviewers by promoting capacity building through mentorship and support groups, ensuring adherence to best-practice integrity standards. Most recently, the journal adopted the Consensus Reporting Items for Studies in Primary Care (CRISP) guidelines, further strengthening the reporting of primary care research.^5^

In addition to its CPD content, SAFP supports registrars and supervisors through regular contributions such as ‘Mastering your Fellowship’ (introduced in 2015),^6^ while the Next5 initiative addresses transitions for early–career family physicians (introduced in 2021).^7^ Most recently, in 2025, the Editorial Fellowship for registrars offers structured immersion in editorial processes and peer review – an investment in future generations that enhances journal capacity and the discipline’s scholarly ecosystem.^8^ Open Forum pieces, editorials and scientific letters provide a platform for timely, practice–focused commentary on issues like equity, access, National Health Insurance (NHI), environmental health, publication ethics and related concerns. These contributions, which fall outside the traditional research agenda but align with the ethos expressed in earlier editorials, confirm that the discipline should continue to emphasise relationship–centred care, uphold rigour in the practice of scientific responsibility and remain responsive to societal needs.

Building on SAFP’s strong legacy in advancing primary care, we remain dedicated to keeping attuned to changing trends. Emerging healthcare needs, technological progress and policy shifts drive us to embrace innovation – whether by championing interdisciplinary research, strengthening ties with primary care practitioners to identify gaps and foster ownership or integrating technology in ways that are ethically sound and socially responsible. The transformative potential of AI tools and clinical decision support systems will only be realised if grounded in the real-world experiences of our practices and communities. Similarly, the NHI presents an opportunity for research-driven clinical governance, enabling us to monitor implementation and drive innovation in the healthcare system.

As we celebrate this 45-year milestone, we look ahead to the half-century mark. We invite our entire community – readers, authors, reviewers and partners – to join in a shared commitment to grow SAFP’s reach and impact strategically. Beyond high-quality submissions, mentorship and advocacy, we specifically welcome your input and ideas for future special collections to help define our strategic focus and ensure SAFP remains the definitive, context-aware voice for family practice research in this evolving healthcare landscape.

## References

[1] Von Pressentin KB, Kaswa R, Murphy S, Nair A. A review of published research in the South African Family Practice-A clarion call to action. S Afr Fam Pract. 2023;65(4): 5777. 10.4102/safp.v65i1.5777 PMC10483307 37526531

[2] Mash B. Changes to the South African Family Practice Journal. South African Family Practice. S Afr Fam Pract. 2019;61(1):a5050. 10.4102/safp.v61i1.5050

[3] Horn L. Promoting responsible research conduct: A South African perspective. J Acad Ethics. 2017;15(1):59–72. 10.1007/s10805-016-9272-8

[4] AOSIS. AI policy summary [homepage on the Internet]. AOSIS; 2025 [cited 2025 Dec 09]. Available from: https://aosis.co.za/legal-centre/publication-policies/#1699617493200-fadce5d6-e96b

[5] Von Pressentin KB, Nair A, Kaswa R, et al. Celebrating our journal’s commitment to strengthening primary health care research. S Afr Fam Pract. 2025;67(1):1–2. 10.4102/safp.v67i1.6139 PMC12067572 40336430

[6] Von Pressentin K. Mastering your fellowship. S Afr Fam Pract. 2015;57(5):45–50. 10.4102/safp.v57i5.4349 PMC8378125 34082562

[7] Van der Bijl C, Nair A, Von Pressentin KB. Next5 – A new South African Academy of Family Physicians initiative (‘You didn’t come this far, to only come this far’). S Afr Fam Pract. 2021;63(4):e1–e2. 10.4102/safp.v63i1.5405 PMC8603212 34797086

[8] Marcus SM, Maluleka MM. Nurturing scholarship: Reflections on the inaugural South African Family Practice Editorial Fellowship. S Afr Fam Pract. 2026;68(1), a6250. 10.4102/safp.v68i1.6250 PMC13058588 41773401

